# Architecture of a Host–Parasite Interface: Complex Targeting Mechanisms Revealed Through Proteomics*[Fn FN2]

**DOI:** 10.1074/mcp.M114.047647

**Published:** 2015-04-30

**Authors:** Catarina Gadelha, Wenzhu Zhang, James W. Chamberlain, Brian T. Chait, Bill Wickstead, Mark C. Field

**Affiliations:** From the ‡School of Life Sciences, University of Nottingham, Nottingham, UK, NG2 7UH;; §Department of Pathology, University of Cambridge, Cambridge, UK, CB2 1QP;; ¶Laboratory of Mass Spectrometry and Gaseous Ion Chemistry, The Rockefeller University, New York, 10021;; ‖Division of Biological Chemistry and Drug Discovery, University of Dundee, Dundee, UK, DD1 5EH

## Abstract

Surface membrane organization and composition is key to cellular function, and membrane proteins serve many essential roles in endocytosis, secretion, and cell recognition. The surface of parasitic organisms, however, is a double-edged sword; this is the primary interface between parasites and their hosts, and those crucial cellular processes must be carried out while avoiding elimination by the host immune defenses. For extracellular African trypanosomes, the surface is partitioned such that all endo- and exocytosis is directed through a specific membrane region, the flagellar pocket, in which it is thought the majority of invariant surface proteins reside. However, very few of these proteins have been identified, severely limiting functional studies, and hampering the development of potential treatments. Here we used an integrated biochemical, proteomic and bioinformatic strategy to identify surface components of the human parasite *Trypanosoma brucei*. This surface proteome contains previously known flagellar pocket proteins as well as multiple novel components, and is significantly enriched in proteins that are essential for parasite survival. Molecules with receptor-like properties are almost exclusively parasite-specific, whereas transporter-like proteins are conserved in model organisms. Validation shows that the majority of surface proteome constituents are *bona fide* surface-associated proteins and, as expected, most present at the flagellar pocket. Moreover, the largest systematic analysis of trypanosome surface molecules to date provides evidence that the cell surface is compartmentalized into three distinct domains with free diffusion of molecules in each, but selective, asymmetric traffic between. This work provides a paradigm for the compartmentalization of a cell surface and a resource for its analysis.

The cell surface is the major point of interaction between unicellular parasites and their surroundings, and is the site for many essential functions such as nutrient uptake, host recognition, and environment sensing. This interface, however, also represents the primary target for host immune attack. To evade adaptive immune defenses, many pathogens (including the causative agents of malaria, Lyme disease and AIDS) use some form of antigenic variation - the expression of a series of immunologically distinct surface proteins (1, 2)). As an exclusively extracellular parasite of the blood, African trypanosomes have made a huge investment in this strategy. In the human-infective species *Trypanosoma brucei*, around ten million copies of a single variant surface glycoprotein (VSG)^1^ form a dense surface coat that protects the parasite against complement-mediated lysis. Periodic switching of the single expressed VSG gene from a vast silent library enables trypanosomes to avoid clearance by the host's adaptive immune response, prolonging infection and increasing the chances of transmission. The monoallelic expression of *VSG* is achieved through tight regulation from telomeric expression sites (ES), with only one from about 20 ES being transcriptionally active at any one time.

For the strategy of antigenic variation to work, the African trypanosome surface coat must be kept free of many essential invariant antigens that might otherwise elicit an immune response. Most of these are thought to be sequestered within a specialized region of the surface membrane at the base of the flagellum called the flagellar pocket (FP). This relatively small membrane domain is the sole site for all endocytosis and exocytosis performed by African trypanosomes, and has the highest rate of endocytosis for any system thus far observed (3, 4). Thus, the FP is a crucial interface between the parasite and host. Unsurprising, disruption of FP function by loss of the associated cytoskeleton or endocytic vesicular traffic is lethal (5–7), highlighting the potential of this host–parasite interface as a therapeutic target.

Our current understanding of FP function and its possible exploitation for therapeutic gain have been significantly inhibited by the paucity of data on its molecular composition. Mining the parasite genome for genes encoding simple characteristics of membrane association is of limited predictive power, as a large proportion of predicted membrane proteins unlikely to be on the cell surface, and in-silico generated datasets are often not amenable to validation studies (for example, the parasite genome is predicted to encode 257 GPI-anchored proteins, 1963 transmembrane proteins, and over 7000 potentially glycosylated proteins, from a pool of only 9202 predicted proteins (genedb.org, v4)). Attempts to purify specific FP components, however, have been hampered by technical difficulties in isolation and thus far none have succeeded in providing validated, high-confidence datasets (8–11).

Only a few validated FP constituents are known to date. The first reported was the heterodimeric transferrin receptor (12) encoded by the ES associated genes 6 and 7 (*ESAG6* and *ESAG7*). Since then, only four more proteins have been specifically localized to the FP of bloodstream-form stages: the haptoglobin–hemoglobin receptor (13), an aquaporin (14), a hypothetical protein identified by proteomics of flagellar fractions (15), and a protein associated with differentiation (16). These components likely represent only a tiny subset of the FP proteome. Here, we address this knowledge gap using a comparative, semi-quantitative approach for the high-confidence identification of cell surface proteins in bloodstream-form *Trypanosoma brucei*. By creating a new genetic toolkit for endogenous locus tagging of membrane proteins, we validate our proteomic set by localization of 25 putative surface molecules of unknown function. As well as demonstrating the location for many novel FP components, we show that individual proteins access different combinations of cell surface membrane domains, and present a bioinformatics analysis of sorting signals. From these data, we propose a new model for the domain organization of the *T. brucei* surface.

## EXPERIMENTAL PROCEDURES

### 

#### 

##### Isolation of Surface Membrane Proteins

**Fig. 1.**
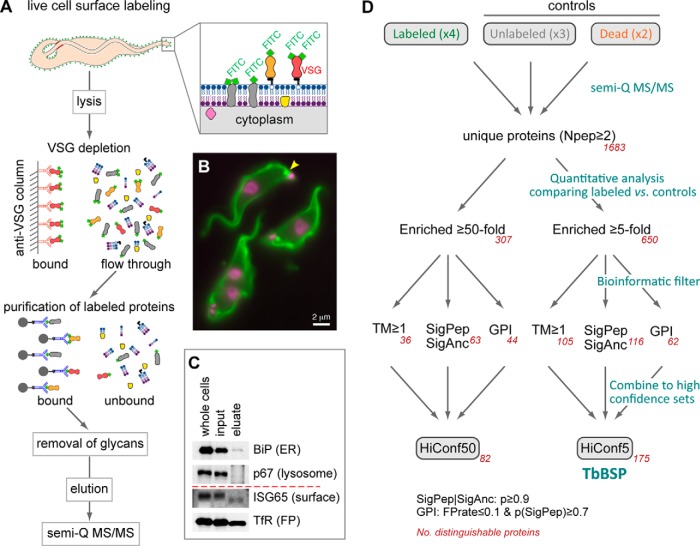
**Workflow of biochemical, semi-quantitative mass spectrometry and bioinformatic methods used to identify putative cell surface proteins.** *A*, Scheme illustrating key steps in purification. *B*, Micrograph of cells following chemical modification with fluorescein (live at 0 °C). Native fluorescence at plasma membrane is predominantly derived from fluoresceinated VSG (which makes up ∼90% of proteins at the parasite surface). DNA has been counter-stained with DAPI (magenta); the FP is indicated by yellow arrowhead. *C*, Immunoblots showing isolation of known surface proteins (ISG65, found on the cell surface and TfR, found in the FP) in the final purified eluate. Note faster migration of deglycosylated ISG and TfR in eluate. Common contaminants from the ER (BiP) and lysosome (p67) are highly depleted in final eluate. *D*, Schematic showing enrichment analysis (for exclusion of contaminants by comparison of labeled samples with controls) and bioinformatic filters (for prediction of membrane proteins features) applied to protein identification to produce “high-confidence” sets. The numbers of unique proteins present in each set are shown in red. The high-confidence set of 175 putative surface membrane proteins enriched 5x in labeled samples is herein referred to as the *T. brucei* bloodstream surface proteome (TbBSP). Experimental replicates of protein isolation from fluorescein-labeled live cells (“Labeled”), unlabeled cells (“Unlabeled”), and fluorescein-labeled material from lysed cells (“Dead”) are indicated between brackets. See Experimental Procedures for details of protein feature prediction, and supplemental Table S2 for bioinformatics filter abbreviations.

We used bloodstream-form *Trypanosoma brucei* Lister 427 expressing VSG221 (BES1/MITat 1.2/VSG427–2/TAR 40), as monitored by immunofluorescence microscopy using an affinity-purified polyclonal antibody anti-VSG221. 5 × 10^8^ mid-log phase cells were harvested by centrifugation and resuspended at 2 × 10^8^ cells ml^−1^ in PBS (10 mm PO_4_, 137 mm NaCl, 2.7 mm KCl, pH 7.5) plus 20 mm glucose. Cells were held on ice while pulsed with 500 μm fluorescein-hexanoate-NHS (referred hereafter to as fluorescein) dissolved in DMSO and HPG buffer (20 mm HEPES pH 7.5, 140 mm NaCl, 20 mm glucose). Pulse duration was 15 min on ice, during which time cells remained actively motile and morphologically normal (as assessed by light microscopy). Fluorescence microscopy showed fluorescein to be exclusively associated to the parasite cell surface (Fig. 1*B*). At the end of this period, unreacted fluorescein was blocked by the addition of TBS (25 mm Tris-HCl pH 7.5, 150 mm NaCl) plus 0.25% w/v glycine, and removed by washing cells in TBS plus 20 mm glucose. Fluorescein-labeled cells were lysed with 2% v/v Igepal CA-630 and 2% w/v CHAPS in the presence of protease inhibitors (5 μm E-64d, 2 mm 1,10-phenanthroline, 50 μm leupeptin, 7.5 μm pepstatin A, 500 μm phenylmethylsulfonyl fluoride, 1 mm EDTA, 1 mm DTT) and 200 μg ml^−1^ DNase I, and centrifuged at 20,000 × *g* for 30 min to separate soluble labeled proteins from the insoluble fraction. To increase identification sensitivity toward less abundant surface membrane proteins, we included a VSG-depletion step by affinity chromatography, for which a polyclonal antibody anti-VSG221 was generated (please see below). The soluble fraction was allowed to bind to 8 mg polyclonal antibody anti-VSG221 conjugated to protein G-Sepharose 4 fast flow (GE Healthcare). Then the soluble fraction partially depleted of VSG was allowed to bind to 30 mg protein G-Dynabeads (Invitrogen) cross-linked to 400 μg polyclonal antibody anti-fluorescein for 1 h, after which period unbound material was collected as flow-through and beads were washed several times in the presence of high salt and detergent (500 mm NaCl, 0.02% v/v Tween-20). Bound proteins were deglycosylated native *on column* with 1000U of PNGase F for 1 h before acid then basic elutions in 0.2 m glycine pH 2.5 and 0.2 m triethanolamine pH 11 respectively. To control for nonspecific binding to anti-fluorescein column, a parallel isolation was carried out with unlabeled cells. To account for possible cell lysis during the surface labeling step, 1 × 10^8^ cells were subjected to hypotonic lysis by resuspension in 20 mm Hepes pH 7.5 in the presence of the protease inhibitors aforementioned for 30 min at room temperature, and then pulsed with fluorescein as above.

##### Mass Spectrometry

Proteins in the final eluate were precipitated with cold acetone, solubilized in Laemmli buffer, and treated with 1 m iodoacetamide to alkylate reduced cysteines. Proteins were resolved by SDS-PAGE using pre-cast gels and standard techniques. Post-electrophoresis gels were stained with SyproRuby (Life Technologies) for imaging, or Coomassie blue for band excision. Mass spectrometry analysis of proteins that were digested in-gel was performed on an LTQ-Orbitrap Velos Pro mass spectrometer (Thermo Scientific). The mass spectrometry proteomics data have been deposited to the ProteomeXchange Consortium via the PRIDE partner repository with the dataset identifier PXD002180.

##### Label-free quantitation of mass spectrometry results

mzXML data files were uploaded onto the Central Proteomics Facilities Pipeline (release version 2.1.1; www.proteomics.ox.ac.uk), which uses Mascot, X!Tandem and OMSSA search engines. The data were searched with the following peptide modifications: fluorescein (K), acetylation (protein N terminus), carbamidomethylation (C), oxidation (M), and deamidation (N/Q). Note that we expect the majority of peptides, even those derived from fluoresceinated proteins, not to contain the fluorescein modification, as only a few lysines in any given surface protein would be accessible. Precursor mass tolerance was set at 20 ppm, MS/MS fragment ion tolerance at 0.5 Da, and number of missed cleavages permitted at 2. Searches were performed against a custom, non-redundant trypanosome protein sequence database combining predicted protein sequences from TREU927 and Lister 427 genomic data (tritrypdb.org), with the inclusion of ES and VSG sequences (17, 18), and containing in total 20,195 entries. The resulting peptide identifications from each search engine were validated with PEPTIDEPROPHET and PROTEINPROPHET and lists compiled at the peptide and protein level. IPROPHET was used to combine the identifications from three search engines and further refine identifications and probabilities. Normalized spectral index quantitation (SINQ) was applied to the grouped meta-searches to give protein-level quantitation between labeled samples and controls (19). All lists of peptide and protein identifications were generated with a probability cut-off corresponding to 1% false discovery rate (FDR) relative to a target decoy database. Only proteins identified with 2 or more spectra were considered for further analysis.

##### Bioinformatics

Signal peptide and anchor sequences were predicted from the first 70 aa of each coding sequence by a stand-alone implementation of SignalP v3.0b (20, 21) using the hidden Markov model methodology, “eukaryotic” settings and thresholds of *p* ≥ 0.9. For GPI-anchor prediction, to reduce false positives, proteins were considered only if they were a PredGPI hit (22) with false-positive rate ≤0.1 and also had SignalP peptide prediction with *p* ≥ 0.7 (because only proteins directed to the endoplasmic reticulum are processed for anchor addition). Transmembrane domains were predicted using TMHMM v2.0c (23, 24).

##### Generation of a Genetic Toolkit for Membrane Protein Localization

A vector for specific tagging of GPI-anchored protein genes, named pSiG, was created by *de novo* synthesis (MrGene, Invitrogen). pSiG contains an epitope tag and fluorescent protein flanked by a signal peptide and GPI-anchor insertion sequences (derived from VSG221) up- and downstream respectively. A derivative for tagging of transmembrane protein genes, pSiS, was created by replacing the GPI-anchor insertion sequence from pSiG with a stop codon generated by annealing two primers. In these vectors, part of the targeted ORF and its UTR, at either the N- or C terminus, is cloned in frame with the epitope tag/fluorescent protein, then the plasmid is linearized for transfection and replacement of the endogenous gene fragments. Hence, the sites for targeting the specific locus are supplied by the user along with the site for linearization. The constructs contain convenient restriction sites on either side of the fluorescent protein/epitope tag for integration of short targeting sequences. Derivatives include nine different fluorescent proteins, two epitope tags and three selection markers. These vectors are available from the authors upon request, and their DNA sequences can be found on the authors' webpage (www.catarinagadelha.com/resources).

##### Endogenous-locus Tagging

ESPs and ESAGs predicted to encode transmembrane proteins were tagged at the C terminus, whereas those predicted to contain a GPI anchor were tagged at the N terminus (because of lack of robustness of prediction algorithms). For N-terminal tagging, PCR amplicons containing ∼200bp from the 5′-end UTR (untranslated region) and ∼200bp from the N-terminal end of the CDS (coding sequence) of interest were cloned together into the XbaI-BamHI sites downstream of the fluorescent protein ORF in pSiG, such that the N-terminal end of the CDS was in frame with the fluorescent protein. For C terminus tagging, PCR amplicons containing ∼200bp from the 3′-end UTR and ∼200bp from the C-terminal end of the CDS of interest were cloned together into the HindIII-AvrII sites upstream of the epitope tag sequence in pSiS, such that the C-terminal end of the CDS was in frame with the fluorescent protein. In the same step, a NotI linearization site was introduced between the UTR and CDS. Integration of these constructs at the targeted endogenous locus results in transgenic lines in which one allele of the CDS of interest contains fluorescent protein at its N/C terminus, but both 5′- and 3′-UTRs are identical to untagged copy. Vectors (∼10 μg) were linearized by digestion with NotI restriction endonuclease and transfected into single-marker bloodstream form *T. brucei* (25) using an Amaxa Nucleofector 2b device, followed by selection of stable transformants with 5 μg/ml hygromycin. Correct integration was assessed by diagnostic PCR from genomic DNA of clonal transformants (not shown) and also immunoblotting of cell lysates separated by SDS-PAGE against a mixture of two anti-GFP monoclonals (7.1 and 13.1; Roche) at 800 ng/ml in 1% w/v skimmed milk in TBS, followed by 80 ng/ml horseradish peroxidase-conjugated goat anti-mouse immunoglobulins.

##### Analysis of Integration into VSG221 Expression Site

Whole-chromosome-sized DNAs were prepared as described elsewhere (26). Agarose-embedded DNA was digested with SmiI endonuclease and subjected to pulsed-field gel electrophoresis in a contour-clamped homogeneous electric field electrophoresis apparatus (CHEF-DR III; Bio-Rad), loading DNA from 1.7 × 10^7^ cells per lane. DNA separation was performed in 1% agarose in TB(0.1)E (90 mm Tris borate, 0.2 mm EDTA, pH 8.2) held at 14 °C for 20 h at 5.2 V/cm with switching time ramped linearly 2–10 s and an included angle of 120°. DNA gels were stained in ethidium bromide and prepared for transfer by UV nicking (80 mJ, 250 nm UV) followed by equilibration in 0.4 m NaOH, 1.5 m NaCl and then transferred to positively charged nylon membrane by capillary transfer in the same solution. After transfer, membranes were neutralized with 0.5 m Tris-HCl (pH 7) and cross-linked (120 mJ, 250 nm UV). Fluorescein-labeled probes were generated by random priming from unlabeled *GFP*, *HYG* and *VSG221* coding sequences. Denatured template DNA (100 ng) were incubated for 5 h at 37 °C with 0.1 mm dATP, dCTP, dGTP, 0.67 mm dTTP, 0.33 mm Fluorescein-dUTP, 2 μm random heptamers and 5 U Klenow fragment. Hybridization was performed overnight in 1% w/v SDS, 5% w/v dextran sulfate, 10% v/v blocking solution (Roche), 750 mm NaCl, 75 mm sodium citrate (pH 7) at 60 °C. Blots were washed to a stringency of 0.1% SDS w/v, 30 mm NaCl, 3 mm sodium citrate (pH 7) at 62 °C. Hybridized probe was detected with anti-fluorescein alkaline phosphatase-conjugated antibody and chemiluminescence. For reprobing, membranes were stripped with hot 0.3% w/v SDS plus 0.3 m NaOH.

##### Protein localization

For analysis of localization of tagged proteins by native fluorescence, cells were harvested from mid-log phase cultures, washed twice in PBS plus 20 mm glucose, allowed to adhere onto derivatized glass slides for 2 min (at density of 2 × 10^7^ cells/ml), fixed for 10 min in 2.5% w/v formaldehyde, counter-stained with 5 μg/ml concanavalin A (ConA; it binds to α-d-mannose and α-d-glucose moieties associated to VSG and possibly other surface proteins) conjugated to tetramethylrhodamine isothiocyanate (TRITC) for 20 min, and mounted in a solution containing DAPI and a photostabilizing agent (1% w/v 1,4-Diazabicyclo(2.2.2)octane, 90% v/v glycerol, 50 mm sodium phosphate pH 8.0, 0.25 mg/ml 4′,6-diamidino-2-phenylindole).

##### Generation and Purification of Polyclonal Antiserum

A fragment encoding residues 27–384 of VSG221 (Tb427.BES40.22) was amplified by PCR from *T. brucei* Lister 427 genomic DNA and cloned in frame into the bacterial expression vector pQE-30 (Qiagen) to allow expression of the coding sequence fragment fused to an N-terminal 6xHis tag. Expression of recombinant protein was induced in M15(Rep4) *Escherichia coli* (Qiagen) and protein was subsequently isolated from cleared, sonicated bacterial lysates by nickel-affinity chromatography by standard methods. 200 μg of recombinant VSG221 was used as immunogen in rabbits. Reactive antiserum was purified by binding to recombinant protein coupled to CNBr-activated Sepharose beads, washed extensively with PBS and eluted with 0.2 m glycine pH 2.5 followed by 0.2 m triethanolamine pH 11. Affinity-purified polyclonal antibodies were dialyzed against PBS and concentrated by ultrafiltration.

##### Immunoblotting of Surface Membrane Protein Isolation Fractions

Immunoblots to test the purification procedure (Fig. 1*C*) were performed with the following polyclonal antisera: anti-ISG65 (kind gift from Mark Carrington, University of Cambridge, UK), anti-TfR (Piet Borst, The Netherlands Cancer Institute, Netherlands), anti-p67 and anti-BiP (James Bangs, University at Buffalo (SUNY).

## RESULTS

### 

#### 

##### Chemical Modification of the Cell Surface

A mechanistic understanding of the interface between African trypanosomes and their mammalian host requires the identification and characterization of the FP molecular composition. As the FP membrane is contiguous with the membranes of both the cell body and the flagellum, it is extremely challenging to isolate pocket proteins through classical cell fractionation procedures. To address this problem, we devised a workflow to specifically isolate cell surface proteins and generate a validated dataset of bloodstream-form cell surface constituents of *Trypanosoma brucei*. Our strategy is summarized in Fig. 1*A* and starts with the chemical modification (fluoresceination) of the surface of live cells held at low temperature (0 °C). Under these conditions, recycling of the surface coat and endocytosis are blocked, but chemical tags are still able to access proteins at both the plasma membrane (∼90% of which being VSG (27, 28)) and also the FP lumen (Fig. 1*B*). Labeled cells were then solubilized and fluoresceinated surface proteins purified by affinity chromatography. The purification method was optimized by a VSG depletion step to increase sensitivity of detection of less abundant surface proteins (Fig. 1*A* and supplemental Fig. S1), and on-column enzymatic removal of N-glycans to improve mass spectrometry identification of glycosylated surface proteins (Fig. 1*A* and supplemental Fig. S1). Finally, to allow more efficient solubilization of membrane proteins and increase dynamic range, the sample was resolved by SDS-PAGE (supplemental Fig. S1), and gel regions subjected to tandem mass spectrometry (GeLC-MS-MS). The final eluate was enriched in an invariant surface glycoprotein (ISG65) that localizes to the cell surface, and the low-abundance transferrin receptor (ESAG6 subunit) which is found in the FP (Fig. 1*C*). High-abundance markers of internal compartments, specifically the abundant luminal ER chaperone BiP and the LAMP-like lysosomal protein p67, were either greatly reduced or undetectable (Fig. 1*C*).

##### Semi-quantitative Comparative Mass Spectrometry Defines a Trypanosoma brucei Surface Proteome

Our surface protein preparation is anticipated to contain many FP proteins as well as those localized more generally to the cell surface and early/recycling endosomes. Many FP components, however, are expected to be present at only tens or hundreds of copies per cell, as seen for the haptoglobin-hemoglobin receptor (13). This necessitates highly sensitive detection, but also the exclusion of inevitable contaminating proteins. To identify proteins specifically enriched in our surface protein preparation, we used a label-free semi-quantitative mass spectrometry approach against two controls: (1) to account for nonspecific binding to affinity chromatography columns, we carried out parallel isolations with unlabeled cells; and (2) to account for cell lysis during the chemical modification, in which the fluorescein tag would access internal proteins as well as those at the surface, a further control was made by labeling hypotonically lysed cells. We then compared the integrated spectral intensities from mass spectrometry of material isolated from labeled *versus* control preparations, allowing for removal of contaminants through testing for signal enrichment in the labeled sample (Figs. 1*D*).

**Fig. 2.**
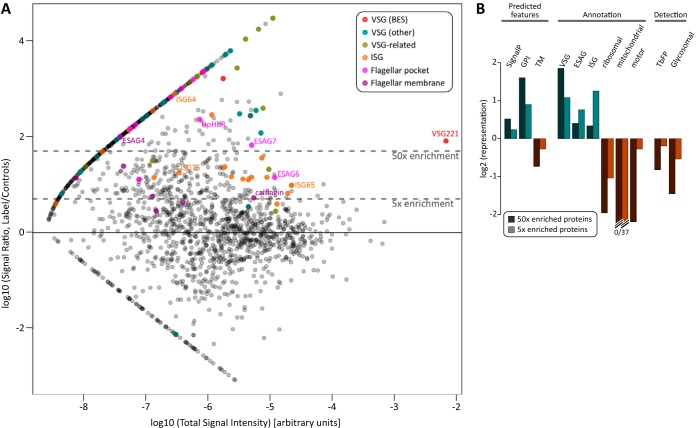
**Identification of surface proteins by comparative label-free semi-quantitative mass spectrometry.** *A*, Enrichment analysis for 1683 unique proteins (integrated spectral intensity) in labeled samples *versus* unlabeled and osmotically lysed controls (see Experimental Procedures for more information). Points represent log10-transforms of total intensity (all replicates, samples and controls) against the ratio of intensity in samples *versus* summed controls. Points representing signal from VSGs (BES copies or from elsewhere in the genome), VSG-related proteins, ISGs, and proteins previously localized to the FP or flagellar membrane are highlighted. *B*, Representation of proteins with select predicted features (SignalP peptide prediction *p* ≥ 0.9; PredGPI false-positive rate ≤0.1; ≥1 predicted TM domain), annotation (word match in description) or those detected in either the *T. brucei* flagellar proteome (30) or glycosomal proteome (31). Representation is the ratio of the number of hits in enriched sets *versus* all uniquely detected proteins.

Across all preparations and replicates, we detected 1683 uniquely distinguishable proteins (each being represented by two or more detectable peptides). The full list of hits and their respective integrated spectral intensities is provided in supplemental Table S1. The most abundant protein in bloodstream-form *T. brucei* cells is VSG (27) and, as expected, VSG221 (MITat 1.2/VSG427–2) expressed from the active ES is detected in all preparations. However, its signal is highly enriched (80×) in labeled samples *versus* controls (Fig. 2*A*), despite being deliberately depleted in these preparations (Fig. 1*A* and supplemental Fig. S1). We also observed, ∼10^3^ times less abundantly, several other VSGs including those in other telomeric ESs, likely representing rare cells in the parasite population which have undergone switch events. Along with these “true” VSGs, a number of VSG-related proteins (transcribed from chromosome internal locations (29)) are also enriched in the preparation, representing the first evidence that this family of proteins is translated in bloodstream form parasites and that they are surface associated.

Our procedure enriches for VSG, several ISGs and proteins known to be localized specifically to the cell surface membrane (Fig. 2*A*). Importantly, low abundance FP proteins, such as HpHbR are also detected in these experiments and are highly enriched (250x) in the labeled preparation. Analysis of the features or annotations of enriched proteins compared with all those detected showed a substantial over-representation of those with predicted signal peptide or glycosylphosphatidylinositol (GPI) anchor, as well those with annotations for “VSG,” “ESAG,” or “ISG” (Fig. 2*B*). Conversely, annotations associated with ribosomes, mitochondrion or cytoskeleton motors are under-represented in the enriched cohort (Fig. 2*B*), as are proteins detected as part of the *T. brucei* flagellar proteome (30) or glycosomal proteome (31). Interestingly, we also find an under-representation of proteins with predicted transmembrane (TM) domains in labeled preparations (Fig. 2*B*). This may suggest that a significant fraction of TM proteins in trypanosomes are expected to be associated with internal membranes, although this may also reflect less efficient chemical modification of multipass proteins with few extracellular lysines (see “Discussion”).

These data show that known surface, FP and flagellum membrane proteins are substantially enriched in our chemically modified preparations. Using enrichment analysis, 307 and 650 uniquely distinguishable protein hits were identified with 50- or 5-fold enrichment in the labeled sample when compared with controls (Fig. 1*D*). However, these sets are unlikely to represent only genuine membrane-associated proteins. To further improve discrimination between true surface proteins and contaminants, we applied a bioinformatic filter to create sets representing only those proteins with predicted signal peptide or signal anchor sequence, GPI-anchor addition sites or TM domains. This is equivalent to intersecting our enrichment data sets with bioinformatic prediction of membrane-association as used by Jackson *et al.* (32), and constitute “high-confidence” sets that have support from both methods. This procedure is also analogous to the approach used to analyze the trypanosome nuclear envelope and identify nuclear pore complex components (33), and here identified 82 or 175 uniquely distinguishable putative surface proteins at 50× or 5× enrichment thresholds respectively (Fig. 1*D*). The full list of these sets is given in supplemental Table S2. These sets represent hits with a high likelihood of being genuine surface proteins, and identified proteins include known FP components, VSGs and ISGs, as well as proteins with predicted function as transporters (Tb427.04.4830, Tb427tmp.02.0630, Tb427.03.4630, Tb427.08.2380, Tb427.08.3620, Tb427.04.4860, Tb427.08.650, Tb427.08.2160), permeases (Tb427.05.3390) and channels (Tb427.10.11680). We herein refer to the 5x-enriched, high-confidence set of 175 putative surface membrane proteins as the *T. brucei* bloodsteam surface proteome (TbBSP).

##### Most TbBSP Proteins are True Parasite Cell Surface Components

**Fig. 3.**
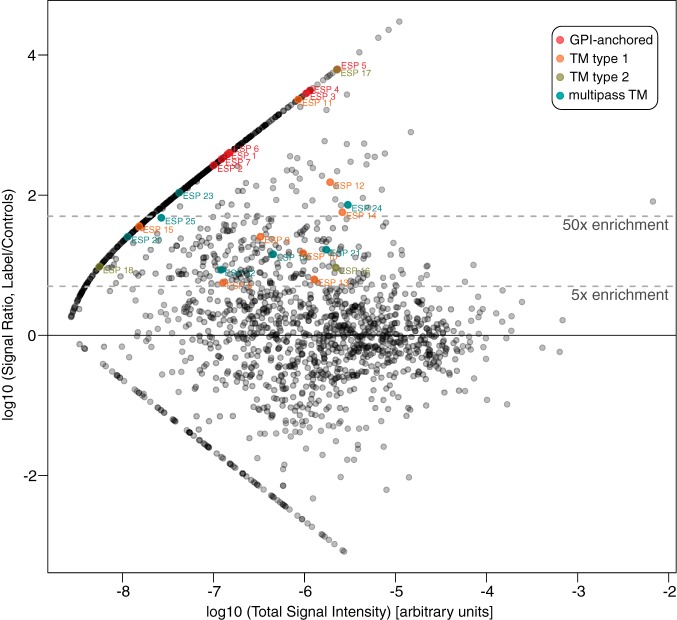
**Enrichment analysis showing proteins of unknown function taken for validation by localization.** Dataset as in Fig. 2, with points representing 25 ESPs highlighted according to predicted protein architecture.

Having demonstrated an efficient enrichment of known FP proteins and related annotation in the labeled dataset, we next sought to robustly test our TbBSP by directly interrogating the cellular location of multiple protein hits of unknown localization, and looking for specific signal at the FP. A set of 25 candidates were selected from the high-confidence sets for further characterization using the following criteria: (1) they were annotated as “hypothetical” proteins for which no functional data had been previously reported for *T. brucei* at the start of this work; (2) they represented the range of general protein topologies detected, *e.g.* predicted GPI-anchored proteins, type I and type II TM proteins, and multipass TM proteins; and (3) they included proteins with enrichment ranging from 5 to >6000 times and spanning >3 orders of magnitude of mass spectrometry signal intensity. Fig. 3 shows the enrichment and architectures of these candidates, and supplemental Table S3 provides their accession numbers and predicted features.

Chimeric proteins were created by integration of tagging constructs at endogenous gene loci. Tagging cell surface proteins is potentially complicated by requirements for signaling sequences at both amino and carboxyl termini, and issues with folding of fluorescent proteins targeted through the ER. To overcome these problems, we created two new series of vectors specifically designed for the endogenous-locus tagging of genes encoding GPI-anchored and non-GPI-anchored sequences containing N-terminal signal sequences. These vectors are called the pSiG and pSiS series, respectively (supplemental Fig. S2) and include the incorporation of a “superfolder” GFP (or derivatives) with improved folding dynamics and greater resistance to the reducing environments encountered in the ER lumen or extracellular space compared with conventional GFP variants (34), plus an epitope tag (HA). The pSiG/pSiS series also include processing signals (trypanosome signal peptide or GPI-anchor addition sequences), providing a means to rapidly and accurately tag surface proteins at either N- or C terminus (supplemental Fig. S2). These vectors provide the correct FP localization of previously analyzed proteins, for example, either GPI-anchored or non-anchored subunits of the transferrin receptor (see Fig. 7). Moreover, the toolkit does not force a non-TbBSP protein (ESAG1) onto the cell surface (supplemental Fig. S2).

The 25 selected genes encoding candidate surface-associated proteins, designated as enriched in surface-labeled proteome (ESP) proteins (ESP1–25), were tagged at their endogenous loci using the vectors described above. Correct integration of the tagging construct and expression of fusion proteins was assessed with immunoblotting of whole-cell extracts (supplemental Fig. 3*A*). Because genes are tagged by integration at the endogenous loci, it is expected that protein expression levels will be close to those for wild-type protein and, consistent with this, different fusion proteins were expressed at different levels. Two tagged proteins (ESP4 and ESP7) did not show a detectable signal on Western blots, and were not pursued further.

**Fig. 4.**
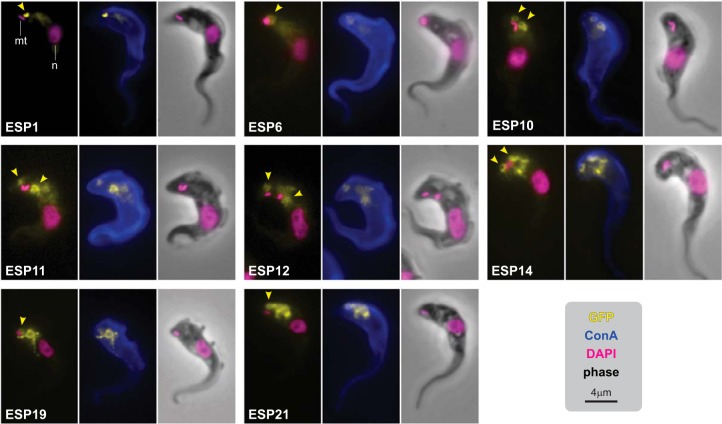
**Localization of surface proteome components at the FP.** ESPs were localized by tagging the gene at the endogenous locus with an ORF encoding superfolder-GFP. Images are representative of the signal distribution observed for each cell line. Yellow: native fluorescence from superfolder-GFP; Blue: concanavalin A counterstain (ConA); Magenta: DAPI. Nuclear (n) and mitochodrial (mt) DNA contents, and FP (yellow arrowhead) are indicated.

For the 23 fusion proteins with detectable expression, 12 were clearly present at the FP membrane as assessed by native fluorescence (Fig. 4 and supplemental Fig. S4). These 12 proteins localized either exclusively to the FP (ESP1, 6, 10 and 11) or in addition to another surface domain - for example, five proteins localized to the FP and endosomal system (ESP12, 14, 19, 21, and 22), whereas ESP8 localized to the FP and the junction between the cell body and the flagellum membranes (the flagellum attachment zone). In addition to these 12 FP proteins, ESP13 and 24 were present across the entire cell surface (FP, flagellum and cell body) and a further four ESPs were predominantly localized to endosomes (ESP5, 9, 15, 20). This is expected, because the endosomal membrane is in constant flux with the cell surface and proteins with clear FP function, such are TfR, maintain a steady-state concentration in early/recycling endosomal compartments (35, 36). Likewise, ISGs are equally distributed between endosomes and FP/cell surface (37). Therefore, these four predominantly endosomal ESPs are likely to be transiently present at the FP, albeit at low abundance, and are thus enriched in our chemical modification procedure. ESP17 and ESP18 were found at both the cell body membrane and an intracellular compartment tentatively interpreted as the lysosome. The remaining five proteins (ESP2, 3, 16, 23, 25) localized elsewhere in the cell and may represent contaminants, although mislocalization because of tagging cannot be excluded (supplemental Fig. S4). Overall, experimental validation by cellular localization of 23 ESPs shows that we have identified 18 novel membrane proteins on the parasite cell surface, the majority of which reside at the FP (exclusively or in combination with another surface membrane domain).

##### Diversification of Parasite Surface Architecture

**Fig. 5.**
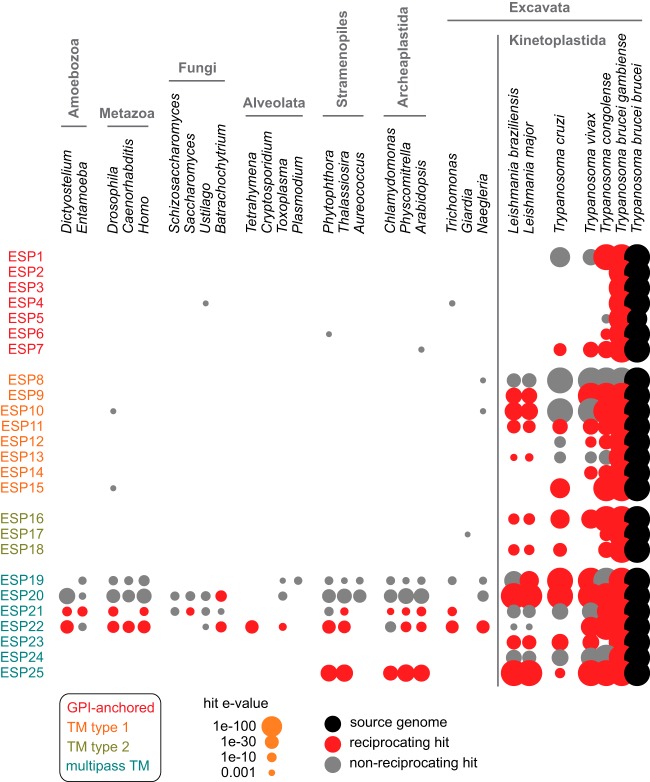
**The distribution of ESPs across eukaryotes.** Conservation was investigated by analysis of BLAST hits in the predicted proteomes of model species from a wide range of eukaryotic lineages. Spot size represents the strength of BLAST hit (e-value). Red shows reciprocal best-BLAST hits between genomes; gray shows non-reciprocating hits.

ESPs at the FP may represent promising therapeutic targets because of their exposure and potential roles in modulating essential parasitic processes, but only if those proteins are sufficiently different to host ones. To map the evolutionary distribution of ESPs, we asked if orthologs could be detected in organisms representing a wide taxonomic diversity of eukaryotes, including humans, and for which complete or near-complete genome sequences were publicly available. Phylogenetic analysis show that most ESPs are specific to African trypanosomes and closely related parasites (Fig. 5). This provides evidence for a lineage-specific architecture for the surface membrane of kinetoplastid cells, reflecting their shared ancestry and biological similarities. Striking, however, was the finding that ESPs predicted to be GPI-anchored are often restricted to *T. brucei*, whereas type I and II TM proteins tend to be conserved in all kinetoplastids (both intra- and extracellular parasites) (Fig. 5). This distribution suggests specific protein evolution to match distinct selective pressures encountered by these parasites, such as mechanisms of survival, host immune invasion and transmission. In contrast, many of the multipass TM ESPs are from families conserved right across eukaryotes (Fig. 5) and, thus, may have arose early in eukaryotic evolution. This likely reflects the expected hierarchy of conservation, with essential transporters being more evolutionarily constrained.

##### Nine out of 12 ESAGs Encode Surface Associated Proteins

The first FP component identified was the TfR previously mentioned, encoded by the expression site-associated genes (*ESAG*s) 6 and 7. There are 12 distinct families of *ESAG*s (*ESAG*1 to 12) that are co-transcribed with the active VSG gene from one of ∼20 telomeric expression sites (ESs). Some or all *ESAG*s may be present in a particular ES (17), and most have chromosome-internal paralogs known as genes-related to *ESAG* (*GRESAG*s). Only a few other ESAGs have been characterized in detail in *T. brucei*: ESAG8 is a protein of unknown function that has been localized to the nucleus (38, 39), whereas ESAG4 is an adenylate cyclase localized to the flagellum membrane (40), and whose activity has been associated with control of parasitemia (41). GRESAG9 is specifically expressed and secreted by the quiescent “stumpy” bloodstream-form stage (42). Finally, an ESAG specific to the subspecies *T. b. rhodesiense* - the serum resistance associated gene, or SRA - confers resistance to a trypanolytic factor associated with the heavy density lipoprotein found in normal human serum (43–46).

**Fig. 6.**
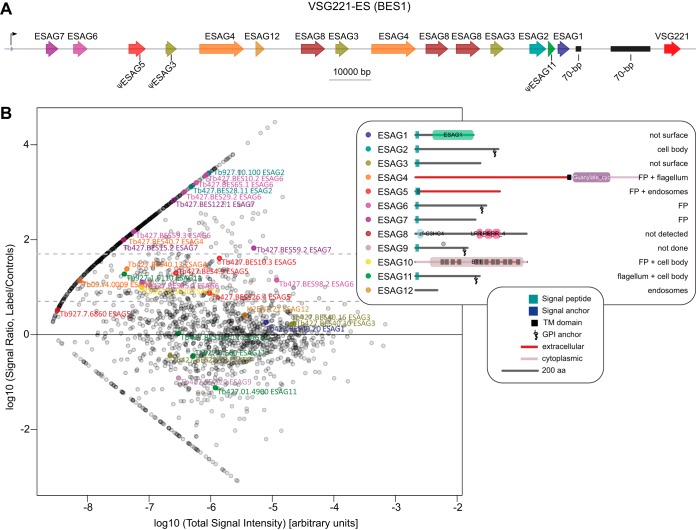
**Identification and validation of ESAG proteins in the TbBSP.** *A*, Structure of the VSG221 expression site (BES1), which is active in cells used in this study. *B*, Distribution of ESAG proteins in the comparative label-free semi-quantitative mass spectrometry. Dataset as in Fig. 2, with points representing members of the 12 ESAG families (including predicted GRESAGs) highlighted. *C*, Protein architectures and localization of the ESAGs. All localization data are from ES copies, except for ESAG5, 10 and 11, which are not present in BES1) for which a detected GRESAG was used. See Experimental Procedures for details of protein feature prediction.

Given that ESAGs are co-expressed with the active VSG during infection, they are believed to play roles in parasite survival in the human host. All but two ESAGs (ESAG8 and ESAG12) are predicted to encode a signal peptide sequence, a GPI-anchor insertion site, or a TM domain, suggesting that they may be associated to the surface membrane or secreted proteins, but for the majority this has not been tested. Significantly, seven ESAGs (ESAG2, 4, 5, 6, 7, 10 and 11) are present in our high confidence datasets (Fig. 6*B*), but the remainder were not. We took this finding, and the genetic tools developed here, as an opportunity to investigate the cellular localization of all ESAGs and to test our surface proteome for false negatives (*i.e.* true surface proteins not detected in our set). We tagged every *ESAG* present in the active ES of bloodstream-form trypanosomes used in this study (BES1, Fig. 6*A*). Only pseudogenes of *ESAG5* and *ESAG11* are present in this ES, and *ESAG9* and *ESAG10* are absent (17). Because chromosome-internal copies of ESAG5, 10 and 11 were highly enriched in our surface proteome, these were also targeted for protein fusions. GRESAG9 has previously been shown not to be expressed in proliferative bloodstream-form parasites (42), and was not pursued here.

**Fig. 7.**
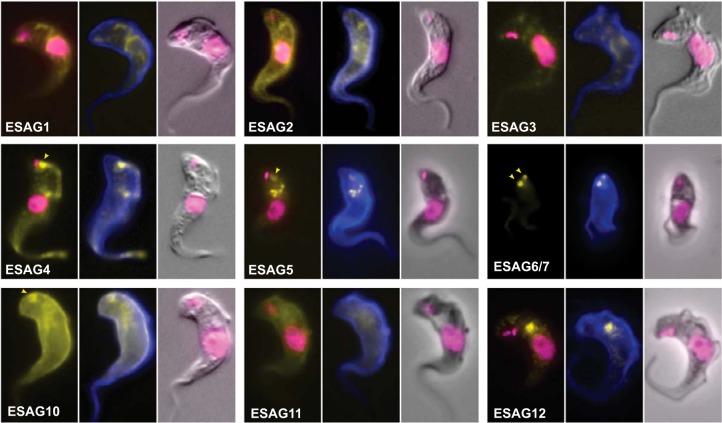
**Most ESAGs encode surface-associated proteins.** 9 ESAGs were localized by tagging the respective gene at the active ES (except for ESAG5, 10 and 11, for which a surface proteome GRESAG was used) with an ORF encoding superfolder GFP and imaged using native fluorescence microscopy. Signal from superfolder-GFP is shown in yellow. Cells have been counterstained with concanavalin A (ConA, blue) and DAPI (magenta). The FP is indicated by yellow arrowhead.

Correct tagging of the active ES copy was confirmed by Southern blotting (supplemental Fig. S5) and ESAG fusion proteins were assessed for correct tagging by immunoblotting (supplemental Fig. 3*B*), and localized by native fluorescence microscopy (Fig. 7). Significantly, all ESAGs detected in our surface proteome localize to the surface membrane. ESAG6/ESAG7 localized to the FP and ESAG4 localized to the FP and flagellum membranes, as previously described (12, 41). Other surface proteome ESAGs localized to the cell body membrane (ESAG2), cell body and FP (ESAG10), cell body and flagellum (ESAG11), or FP and endosomes (ESAG5). With respect to those ESAGs not detected or not enriched in our surface proteome, ESAG12 was detected in endosomes, consistent with being also at the surface at low levels and/or recycling through the endomembrane and surface compartments. ESAG8 was expressed at levels close to the limit of detection by immunoblot when tagged at either end of the endogenous ES copy, and was undetectable in localization experiments. Importantly, ESAG1 and ESAG3 - which contain signal sequences suggestive of possible surface-association, but which were not enriched in the surface proteome—did not localize to the cell surface when tagged. These data demonstrate that (1) tagging with our vectors does not cause non-TbBSP proteins to mislocalize to the surface and (2) ESAG1 and 3 are unlikely to be surface-associated. Hence, of the 12 ESAG proteins, nine are shown to be surface-associated or secreted, five of which present at the FP membrane, clearly arguing for direct roles in host–parasite interactions by virtue of being exposed to the host environment.

##### Protein Localization Suggests Distinct Functional Membrane Domains Maintained by Selective Barriers

The trypanosome surface can be conceptually divided into three regions of contiguous membrane: the FP, the flagellum membrane and the cell body. Our localization data, using the same tag with 14 hypothetical proteins and 6 ESAGs that clearly target the cell surface membrane, allowed us probe for the existence of these or other membrane domains with the largest set of trypanosome surface proteins systematically tested to date. Individual proteins in our sets were found to be restricted to any one of these domains or to combinations of them (Fig. 4 and supplemental Fig. 4), suggesting that the three regions indeed act as specialized domains of surface membrane, divided by selective barriers. Notably, we found only one example of sub-localization within a region (for ESP8), indicating that most proteins have free diffusion within each of the surface membrane domains.

**Fig. 8.**
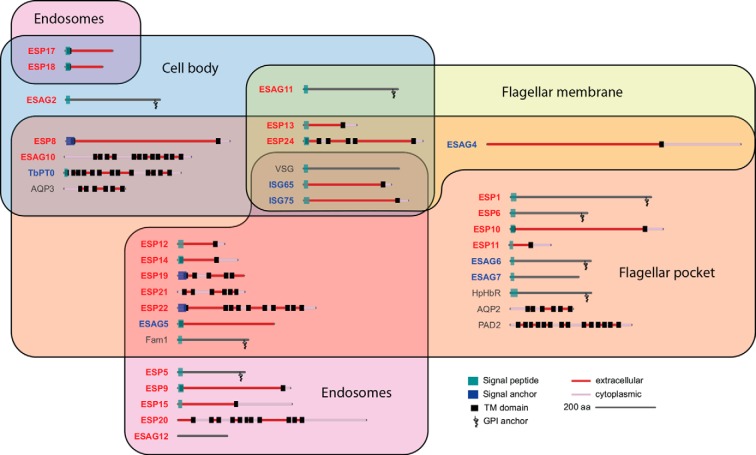
**Domain architecture for surface-associated proteins in *T. brucei*.** Names of proteins which were localized as part of this study are emboldened (red: localized for the first time; blue: also localized in other studies). Data from proteins that have been previously localized by equivalent tagging methods (only) are also shown for comparison (references included in supplemental Table S3). See Experimental Procedures for details of protein feature prediction.

A polarized distribution of ESPs and ESAGs implies intrinsic protein-sorting signals governing location on the cell surface. We therefore analyzed this set for the presence of common sequence motifs or structure that might regulate such sorting; However, no simple correlation between cellular localization and protein architecture emerged. For example, predicted GPI-anchored proteins were not all restricted to the FP, nor were type I TM proteins restricted to the cell body membrane (Fig. 8). Furthermore, motif elicitation analysis (MEME) detected no common motifs among ESPs and ESAGs with shared localization (data not shown). This suggests that protein topology alone may not be the primary determinant of surface domain segregation in *T. brucei*, and more complex interactions are at play.

## DISCUSSION

### 

#### 

##### A Surface Proteome for African Trypanosomes

Here we describe a high-confidence, validated surface proteome for the major host form of African trypanosome parasites. This was achieved through a novel biochemical preparation in which the use of fluorescein was one of several steps optimized to increase both the specificity and sensitivity of our approach. Cell surface proteomic studies of other human pathogens, as well as mammalian cells, have frequently used the biotin-avidin based system to isolate plasma membrane proteins (47–50). In initial experiments we too used sulfo-NHS-biotin to chemically modify the surface of live trypanosomes. However, following affinity chromatography with streptavidin, we found the specificity of the approach was compromised by high background from control (unlabeled) cells, which could not be removed even on extensive washing. This may be a product of the parasite's intrinsic biochemistry: trypanosomatids (except those harboring bacterial endosymbionts) are unable to synthesize biotin (51); but this vitamin is an essential requirement for cell growth (52), and known to be incorporated into endogenous proteins (53). To avoid contamination with endogenously biotinylated parasite proteins, we abandoned biotin as a chemical tag, and moved to fluorescein labeling combined with an antigen–antibody purification system. Fluorescein is cell-impermeable, ensuring that only surface membrane proteins from intact cells are labeled by covalent modification of accessible lysine residues, and antigen-antibody columns can be washed to high stringency. Fluorescein also has an advantage that it can be followed visually or by fluorimetry during preparations. Using this approach, we have developed here a strategy for the identification of surface-exposed membrane proteins, which in trypanosomes isolates proteins that, at steady-state, reside at the FP, early/recycling endosomes, flagellum and cell body membranes.

Our surface proteome was extracted from a specific biochemical preparation coupled with comparative semi-quantitative mass spectrometry and bioinformatic filters. The bioinformatic methods used decrease the risk of contaminants in the defined TbBSP in a manner analogous to those used to describe the high-quality set of nucleoporins that compose the trypanosome nuclear pore complex (33). In that study, an initial set of 757 mass spectrometry hits was reduced by removing 448 contaminants on the basis of functionally unrelated sequence homology and gene annotations (*e.g.* ribosomal, endoplasmic reticulum and cytosolitc proteins). The remaining 309 proteins were informatically filtered for features associated with known nucleoporins (such as functional motifs, molecular weight and predicted secondary structure) (33). Here we filtered our experimental data for sequences that predict targeting to the endoplasmic reticulum and membrane anchoring (either via a GPI anchor or a transmembrane domain).

The contrasting approach of interrogating the entire genome sequence for cell surface localization on the basis of bioinformatic prediction of membrane-association is not applicable to our question because ∼15% of the parasite's predicted proteome (1465 proteins) have such features. To define a predicted cell surface phylome, Jackson and colleagues (32) combined this approach with sequence clustering to look more specifically at those putative membrane-associated proteins in multigene families. Of the 50 CSP families present in *T. brucei*, 20 are detected in the surface proteome (supplemental Fig. S6). Particularly well-represented are Fam10 and Fam79 (supplemental Fig. S6), which comprise proteins of unknown function for which we present the first experimental evidence. For example, of the 7 members in Fam10, five were detected in our dataset, and we have demonstrated the surface association of one (ESP17). Significantly, however, the majority of proteins (63%) in the surface proteome are not part of multigene families (and hence not part of the CSP), yet are *bona fide* surface-associated proteins according to our validation experiments (12 out of 18 ESPs). This highlights the strength of our joint approach of sensitive, semi-quantitative detection and bioinformatic filtering.

##### Extent of the Surface Proteome

The surface proteome includes almost all previously characterized surface proteins for *T. brucei* (albeit rather few in number), as well as hypothetical proteins with predicted function as receptors, transporters, channels and others. These data suggest that the overall coverage of surface proteins in our high confidence set is broad, although it is to be expected that it will not be complete. A natural limitation of our approach is that it only derivatizes surface components with regions of modifiable polypeptide chain exposed to the extracellular space. This excludes proteins solely associated with the cytoplasmic side of the membrane. Hence, proteins modified by N-terminal palmitoyl- or myristoylation (such as for the flagellum calcium-binding protein calflagin (54)) are not expected to be present. Such proteins were not the focus of this work, as our primary objective was to gain knowledge of the molecular components exposed at the host–parasite interface.

A more significant cohort of proteins that may be underrepresented in our surface proteome are those with few exposed extracellular lysine residues. This may explain why aquaporins 2 and 3 (shown to localize to the FP and cell body membranes of *T. brucei* (14)) are not present in the TbBSP. Neither is a putative calcium channel protein (FS179/Tb927.10.2880) localized to the region of flagellum attachment to the cell (15)). These proteins are multipass TM proteins and are predicted to have limited sequence on the extracellular side of the membrane (*e.g.* aquaporins have only three lysines predicted to be extracellular, which may or may not be accessible to fluoresceination depending on the folding of the protein). A number of transporters and channel-like proteins are present in the surface proteome (10/175 proteins in total)—and validation showed that five detected multipass TM proteins are indeed surface-associated—but it is noteworthy that proteins with predicted TM domains were under-represented in our preparations (Fig. 2).

##### Confidence of Surface Prediction

We believe that a specific strength of the present work is the robust validation. Alongside bioinformatic support, we also developed a genetic toolkit to test a subset of 25 candidates for FP/surface localization. The majority were true surface components (14 out of 23 detectable fusion proteins were present at the cell surface, four found predominantly in endosomal compartments that are likely to cycle to the surface in small amounts, whereas five localized elsewhere in the cell). This suggests that our surface proteome contains relatively few false-positives (∼22% at the >5× threshold, and likely far fewer at greater enrichment values).

A number of ESAGs were present in our surface proteome and were localized to the cell surface when tagged, compared with only 1 out of 4 (ESAG12) not present in the TbBSP (despite containing sequence characteristics that might have suggested surface proteins). Although this is only a small set, it does indicate that the levels of false-negatives in our analysis (*i.e.* proteins that should have been detected, but were not) is also proportionally low. It is improbable that our surface proteome contains all proteins resident at the parasite surface, but results from localization of hypothetical proteins and ESAGs indicate a high confidence for the 175 proteins identified herein.

One issue with the interpretation of localization data for ESPs and ESAGs is in defining where the cell surface ends. Most of the proteins tested were detectable by light microscopy at locations in the cell consistent with being the FP, flagellum or cell body membranes. However, the plasma membrane is highly dynamic and is in constant exchange with components of the endosomal system. In trypanosomes, TfR, ISG and VSG are all present in endosomes as well as at the cell surface. It is thus possible that some of the TbBSP proteins not localized to cell surface domains are still molecules that are found transiently or in low abundance at the cell surface. In mammalian cells it is common to find many proteins cycling between the cell surface and early/recycling endosomes, but proteins as “deep” as those found in lysosomes have also been observed on the surface (55–57). African trypanosomes too have a transport route for newly synthesized lysosomal membrane glycoproteins to exit the Golgi and reach the lysosome via the FP (58), though the lysosomal marker p67 may take a direct route that bypasses the FP membrane (59). Hence, it may be biologically meaningful that proteins such as ESP15 (a type I TM protein that localized to the lysosome) is in the surface proteome, whereas p67 (also a type I TM glycoprotein) is not.

##### Membrane Domains and Domain Maintenance

The few surface proteins analyzed to date suggest the existence of at least three biochemically distinct domains across contiguous membranes, and emphasize the idea that individual proteins can access one or more domains on the cell surface. For example, TfR is restricted to the FP and endosomes, the adenylate cyclase encoded by ESAG4 is present at the FP and flagellum, and VSG is distributed across the entire surface membrane and endosomal system. The work here considerably expands these observations, showing that 8 ESAGs and 14 proteins of unknown function localize to one or more of three separate membrane domains: the FP, the flagellar membrane, the cell body. Moreover, these proteins do so in all possible combinations (with the exception of flagellar membrane alone, which was not observed).

Our results support a model whereby trypanosome surface organization is determined by control of access to any of three membrane domains. The finding that only one surface protein (ESP8) showed evidence of sub-domain localization suggests that diffusion within each domain is essentially free for most components. However, selective diffusion barriers or very rapid transfer systems exist between these domains. Because newly synthesized proteins are delivered to the FP, most combinations could be produced by the “opening” of symmetrical barriers at either the base of the flagellum (to access the flagellar membrane) or distal end of the FP (to access the cell body membrane). Nonetheless, the existence of proteins that are enriched in at just the cell body (ESP17, ESP18 and ESAG2) or cell body plus flagellar membrane (ESAG11) suggests that for at least some of the surface proteins the barriers or protein movement must be asymmetric.

This model raises major questions with regards to the mechanisms underlying protein sorting and retention in African trypanosomes, and elucidating such mechanisms in any cell type remains a formidable challenge. We considered that common motifs within the primary sequence might be used to target ESPs and ESAGs to their respective domains or enable them to cross specific domain boundaries, but simple common signals were not found in our analyses. It is also clear that gross protein architecture (*e.g.* GPI-anchor, type I TM, etc.) is not predictive of domain localization, suggesting that the signals are encoded by more complex or protein-specific cues.

The barriers to protein movement on the cell surface are likely to be contained in the structural features described at the boundaries between the domains—the rows of intramembrane particles seen by freeze-fracture electron microscopy forming the ciliary necklace at the junction of the flagellum and FP membranes, and the junction of the FP and neck membrane (60). The molecular identity of these particles remains unknown, but a morphologically similar configuration identified at the base of the mammalian primary cilium requires the GTPase septin for retention of receptors in that organelle (61). Alternatively, lipid composition, particularly that able to accommodate the geometric constraints of highly curved membrane sections (like that at the junction of the flagellum and the FP) could act as barriers to protein movement or as targeting signal. The distribution pattern of membrane probes and GPI-anchored YFP between the ciliary and plasma membranes are consistent with lipid composition operating in this manner (62).

For two trypanosome membrane proteins, lateral movement between surface domains appears to be dependent on protein abundance as well as identity. Over-expression of a membrane-bound acid phosphatase predominantly found in endosomes causes it to re-distribute over the whole cell surface (36). In a similar manner, TfR in excess of normal levels is no longer retained in FP and endosomes, and escapes to the entire cell surface (35). The relevance of such artificial over-expression to endogenous protein targeting is uncertain, but trypanosomes grown in serum with low-affinity transferrin compensate by up-regulating the expression of TfR which, in turn, escapes the FP (35). However, it is clear that surface domain targeting in trypanosomes must be more complex than just a saturable mechanism of FP retention, as has been proposed for TfR, because we observe proteins with localizations specific to each individual domain, and combinations thereof - including proteins excluded from the FP (*e.g.* ESAG2), from most of the cell body (*e.g.* ESP8) or from the flagellum membrane (*e.g.* ESAG10).

##### Unraveling the Host–Parasite Interface

With a cell body entirely covered by ten million copies of a single glycoprotein, cellular functions that would normally occur at the plasma membrane of a typical eukaryotic cell are here concentrated at the FP of trypanosomes. The restriction of endocytosis and secretion to a focal point on the parasite surface allows for invariant receptors, channels and transporters, and other signaling molecules to be sequestered in an environment that is protected from the attention of host defenses, whereas the cell body membrane is mostly denuded of those proteins. Sitting at the critical interface between host and parasite, it is surprising that so few components of the FP have been described prior to this study. The essential nature of receptors such as TfR and HpHbR highlights the FP as an area of vulnerability that could be exploited in a therapeutic context. Our work has expanded this portfolio to 12 novel FP components with proven localization and identifies a total of 175 in the surface proteome, >50% of which are estimated to also be FP proteins. Importantly, 60% of surface proteome components cause a significant loss-of-fitness when knocked down individually (50/83 genes covered in a large-scale RNAi library screen (63)) compared with 42% for all genes (*p* = 0.001), showing that the TbBSP is notably enriched in genes essential for growth in the bloodstream. Because these proteins are mostly parasite specific and exposed to the extracellular space, our surface proteome is a potential source of drugable targets for disease treatment and control.

The high-confidence surface proteome described here greatly increases our knowledge of the trypanosome surface, and provides a significant resource against which hypothesis about membrane protein sorting and retention might be tested. Moreover, the methods we described are widely applicable to the study of cell membrane composition in human pathogens in general; whereas the surface compartmentalization is significant for understanding trypanosome biology and an important paradigm for surface organization in other systems.

## Supplementary Material

Supplemental Data

## Footnotes

^1^ The abbreviations used are:

VSG: variant surface glycoprotein
FP: flagellar pocket
ESP: enriched surface-labeled protein
UTR: untranslated region
TM: transmembrane
GPI: glycosylphosphatidylinositol.
