# Supplementary material for: Architecture of a Host–Parasite Interface: Complex Targeting Mechanisms Revealed Through Proteomics
Source: Mol Cell Proteomics. 2015 Apr 30;14(7):1911–26. doi: 10.1074/mcp.M114.047647 (PMC4587319; doi:10.1074/mcp.M114.047647)
Supplement: Supplemental Data [file supp_14_7_1911__index.html]

Architecture of a host-parasite interface: complex targeting mechanisms revealed through proteomics — Architecture of a host-parasite interface: complex targeting mechanisms revealed through proteomics — Architecture of a Host–Parasite Interface: Complex Targeting Mechanisms Revealed Through Proteomics — Proteomic Definition of a Host–Parasite Interface — Supplemental Data 

# Architecture of a Host–Parasite Interface: Complex Targeting Mechanisms Revealed Through Proteomics

## Supplemental Data

**Files in this Data Supplement:**

- Supplemental Figure Legends - Supplemental Figure Legends
- Supplemental Figure 1 - Supplemental Figure 1
- Supplemental Figure 2 - Supplemental Figure 2
- Supplemental Figure 3 - Supplemental Figure 3
- Supplemental Figure 4 - Supplemental Figure 4
- Supplemental Figure 5 - Supplemental Figure 5
- Supplemental Figure 6 - Supplemental Figure 6
- Supplemental Table1 - Supplemental Table1
- Supplemental Table2 - Supplemental Table2
- Supplemental Table3 - Supplemental Table3
