# Supplementary material for: Architecture of a Host–Parasite Interface: Complex Targeting Mechanisms Revealed Through Proteomics
Source: Mol Cell Proteomics. 2015 Apr 30;14(7):1911–26. doi: 10.1074/mcp.M114.047647 (PMC4587319; doi:10.1074/mcp.M114.047647)
Supplement: Supplemental Data [file supp_M114.047647_mcp.M114.047647-1.pdf]

SUPPLEMENTAL LEGENDS

Supplemental Figure 1. Isolation of fluorescein-labelled surface membrane proteins. Fractions from the purification workflow depicted in Figure 1 were resolved by SDS-PAGE and stained with SyproRuby. The gel shown is representative from experimental replicates. From left to right: whole cell lysate; insoluble and soluble fractions following fluoresceinated cell lysis; eluate and flow-through following VSG depletion of soluble fraction (boxed red); unbound, washed and bound materials from VSG-depleted fraction subjected to anti-fluorescein column; flow-through from in-column deglycosylation treatment (major band represents PNGase F); acid and basic elutions and retained material from anti-fluorescein column. $3x10^6$ cells equivalents were loaded on each lane. Lanes from first and second acid elutions from anti-fluorescein columns were excised and subjected to mass spectrometry identification.

Supplemental Figure 2. A genetic toolkit for membrane protein localization. A) Schematic representation of two vectors from the toolkit for tagging of trypanosome surface proteins. These vectors provide the means to tag surface proteins at either the N- or C-terminus by expression from their endogenous loci. Vectors allow incorporation of: "superfolder" GFP, hemagglutinin epitope tag (HA), and trypanosome signal peptide or GPI-anchor addition sequences. B) Cartoon depicting tagged GPI-anchored (left) or transmembrane (right) proteins. GPI-anchored proteins are illustrated in orange, along with the GPI anchor (black), transmembrane proteins (grey), palmitoylated/myristoylated proteins (pink), and peripheral proteins (yellow). C) Surface proteome toolkit tagging vectors do not force a non-surface GPI-anchored protein (ESAG1) onto the cell surface. Signal from superfolder-GFP is shown in yellow. Cells have been counterstained with concanavalin A (ConA, blue) and DAPI (magenta).

Supplemental Figure 3. Immunoblot of whole-cell extracts from ESP- (A) and ESAG-tagged cell lines (B). $2.5x10^6$ cells equivalents were loaded on each lane. Fusion proteins were

detected with monoclonal antibodies against GFP. Nitrocellulose membrane stained with Ponceau S shown as loading control.

Supplemental Figure 4. Validation of surface proteome components. Cellular localization for 23 ESPs from high-confidence surface proteome sets by endogenous-locus tagging and native fluorescence microscopy. Images are representative of the signal distribution observed for each cell line. Signal from superfolder-GFP is shown in yellow. Cells have been counterstained with concanavalin A (ConA, blue; except for ESP15) and DAPI (magenta). Nuclear (n) and mitochodrial (mt) DNA contents, and FP (yellow arrowhead) are indicated on ESP1 panel.

Supplemental Figure 5. Confirmation of tagging vector integration in VSG221 expression site (BES1). DNA from ESAG-tagged and parental cell lines was digested with SmiI endonuclease, which cuts upstream of the BES1 promoter, producing a ~53 kb fragment in untagged cells. Hybridization of separated DNA to either *GFP* + *HYG* and *VSG221* probes is shown. Integration of the tagging vector into BES1 increases the size by ~5kb. Small differences in BES1 size between cell lines are attributable to variability in ES-associated repeats.

Supplemental Figure 6. Representation of cell surface phylome (CSP) families (32) in the surface proteome. Blue bars represent the number of detected members from each *T. brucei* CSP family detected; red bars denote the percentage representation of each CSP family.

Supplemental Table 1. Table of all proteins identified by mass spectrometry over 4 independent experiments. The sum of all fragment ion intensities that could be attributed to each individual proteins were used for semi-quantitative analysis of enrichment.

Supplemental Table 2. Table listing all proteins contained in the bloodstream-form *T. brucei* bloodstream surface proteome (TbBSP).

Supplemental Table 3. Table listing the gene IDs (tritrypDB.org accession numbers) plus additional information for the 25 ESPs and 10 ESAGs tagged in this study.
