# Supplementary figures and images for: Architecture of a Host–Parasite Interface: Complex Targeting Mechanisms Revealed Through Proteomics
Source: Mol Cell Proteomics. 2015 Apr 30;14(7):1911–26. doi: 10.1074/mcp.M114.047647 (PMC4587319; doi:10.1074/mcp.M114.047647)

## Supplemental Figure 1

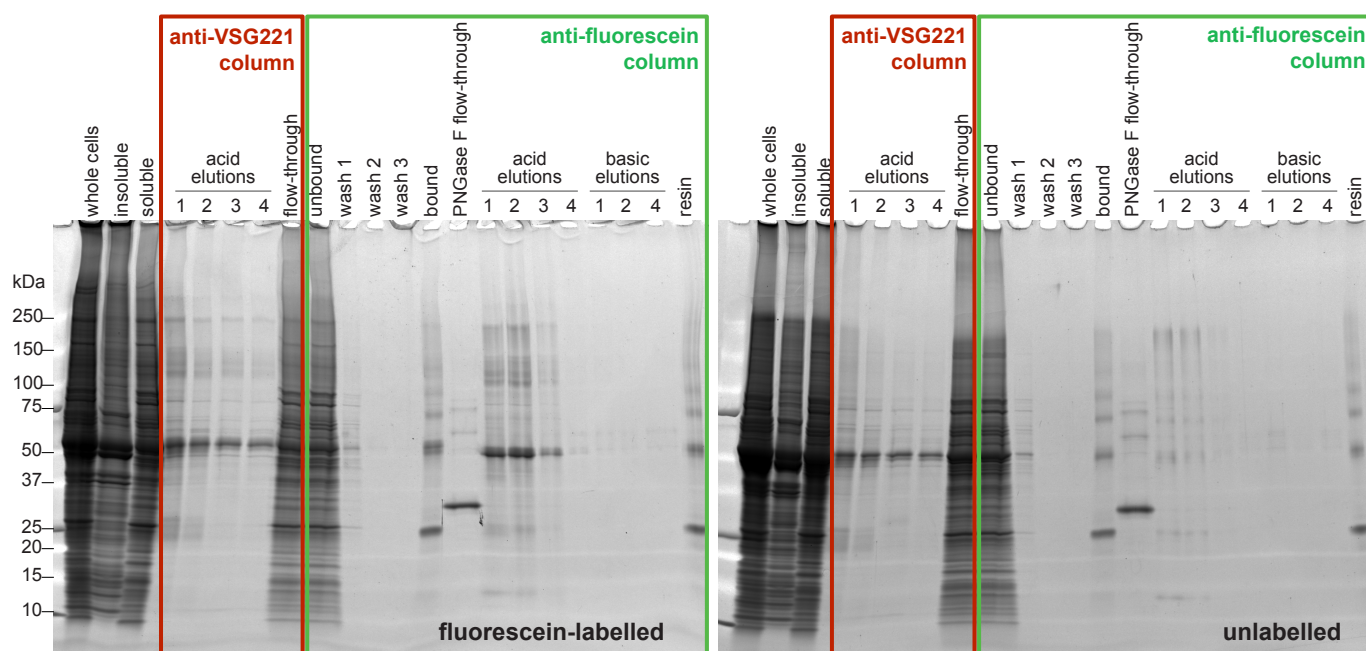

Supplement: Supplemental Data [file supp_M114.047647_mcp.M114.047647-2.pdf]

## Supplemental Figure 2

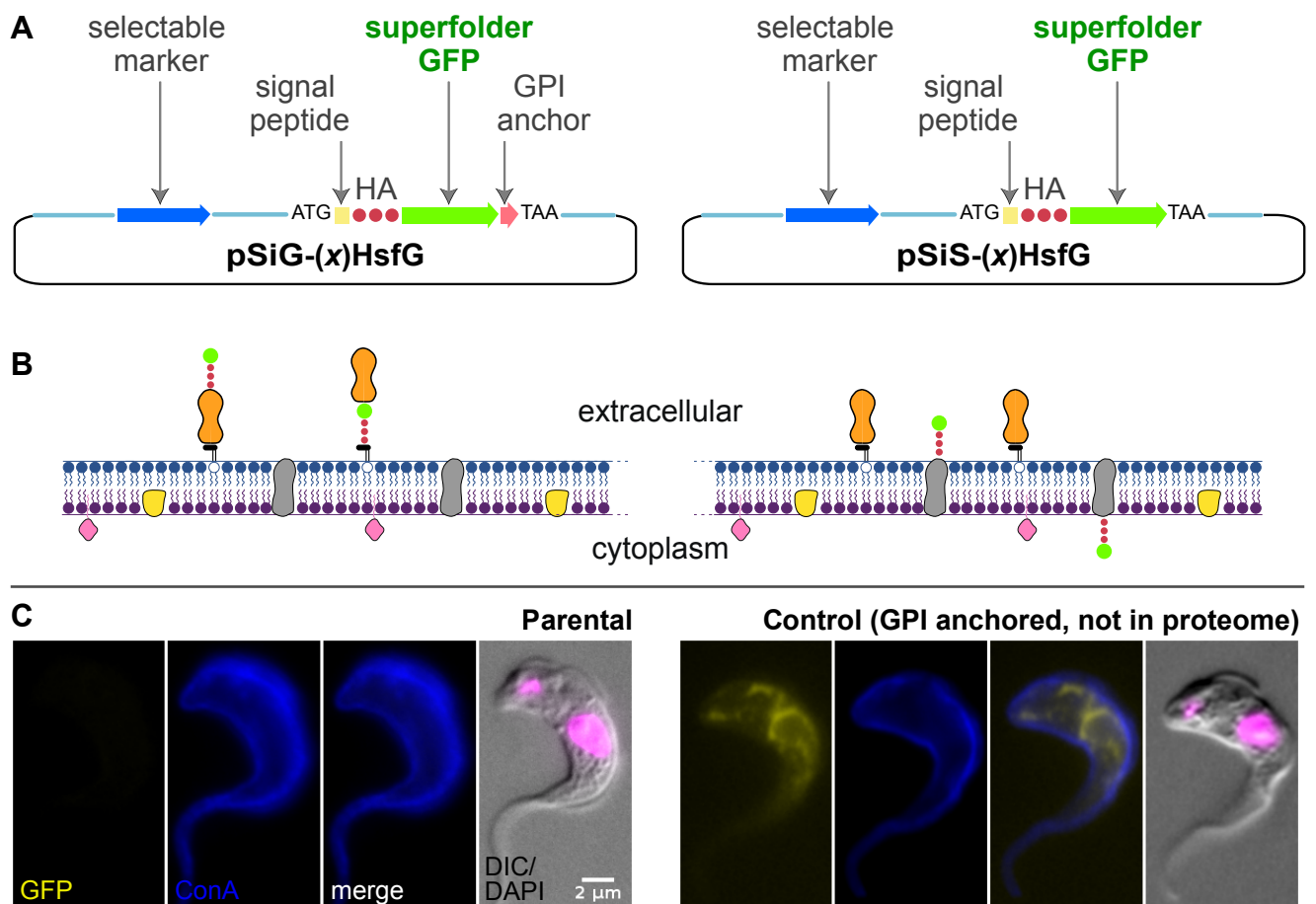

Supplement: Supplemental Data [file supp_M114.047647_mcp.M114.047647-3.pdf]

## Supplemental Figure 3

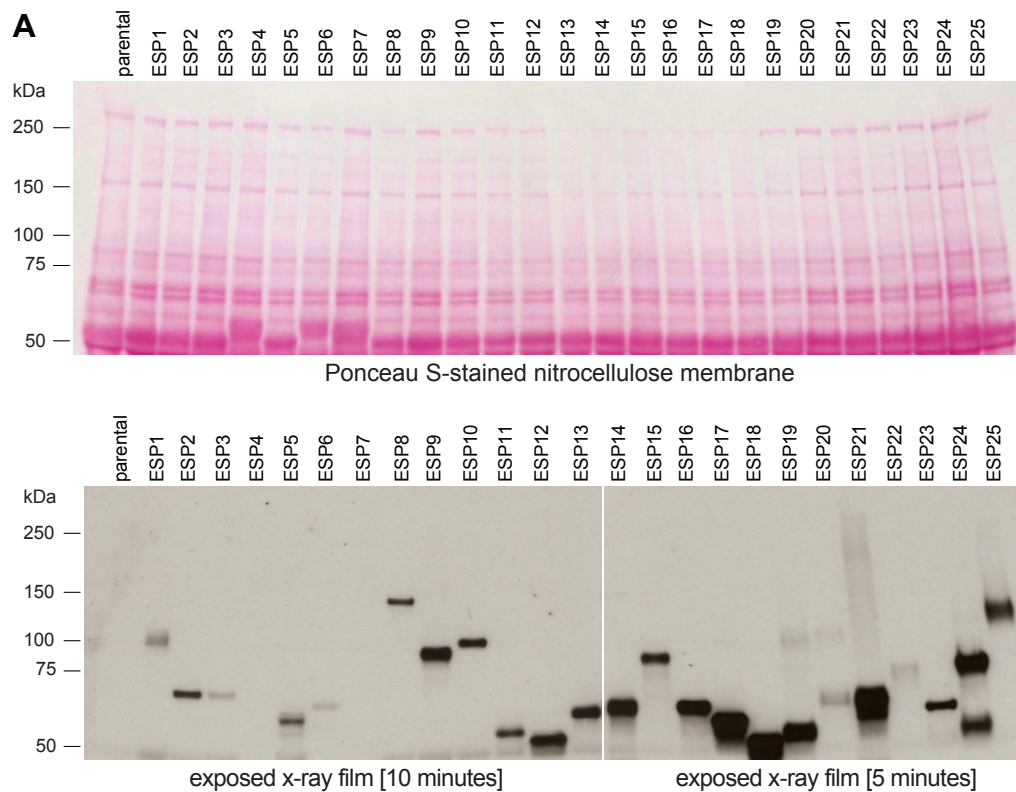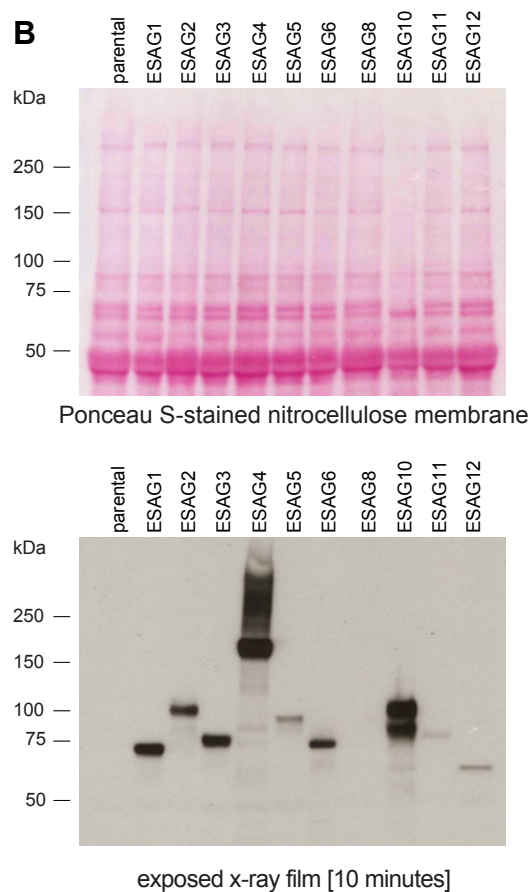

Supplement: Supplemental Data [file supp_M114.047647_mcp.M114.047647-4.pdf]

## Supplemental Figure 4

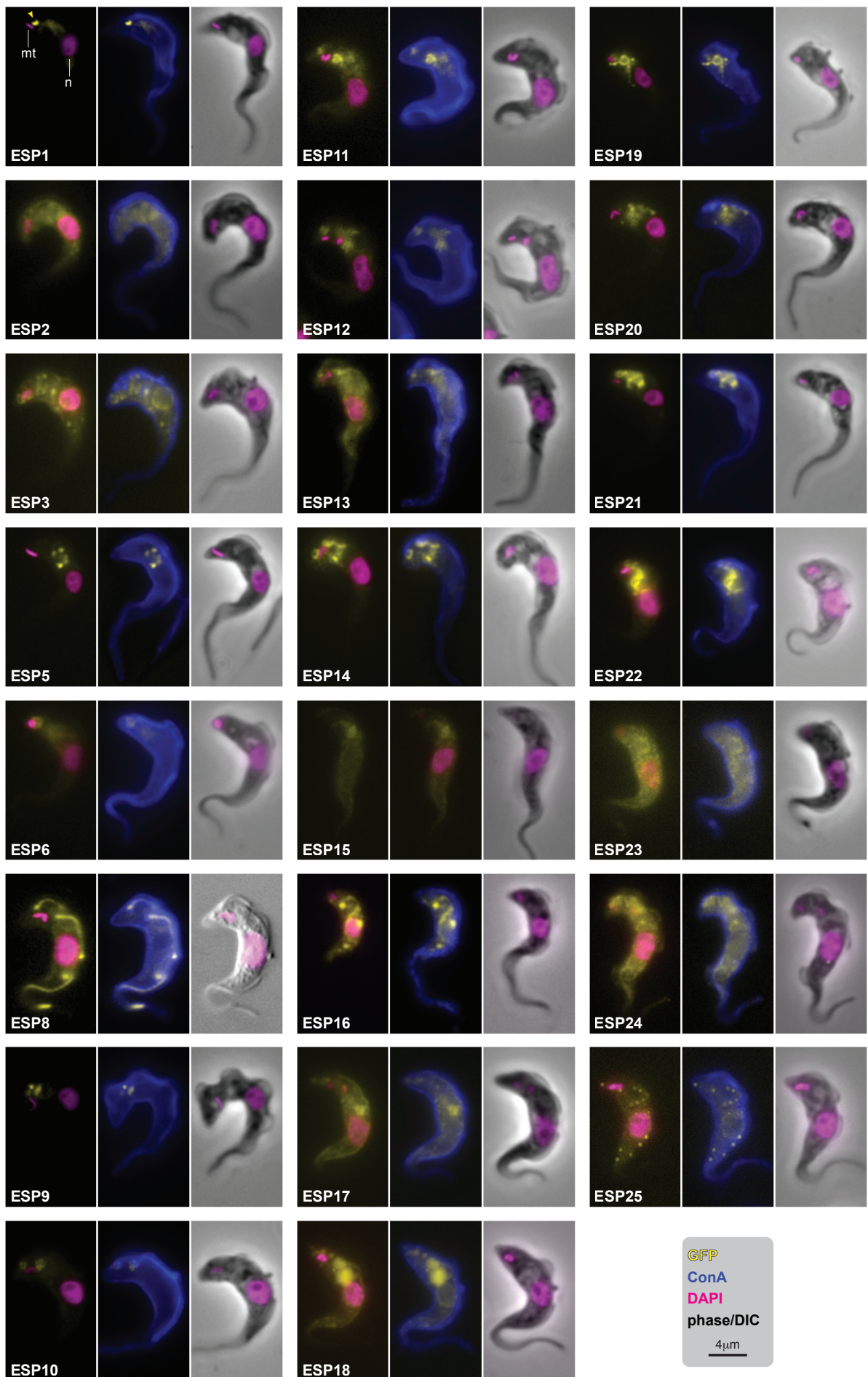

Supplement: Supplemental Data [file supp_M114.047647_mcp.M114.047647-5.pdf]

## Supplemental Figure 5

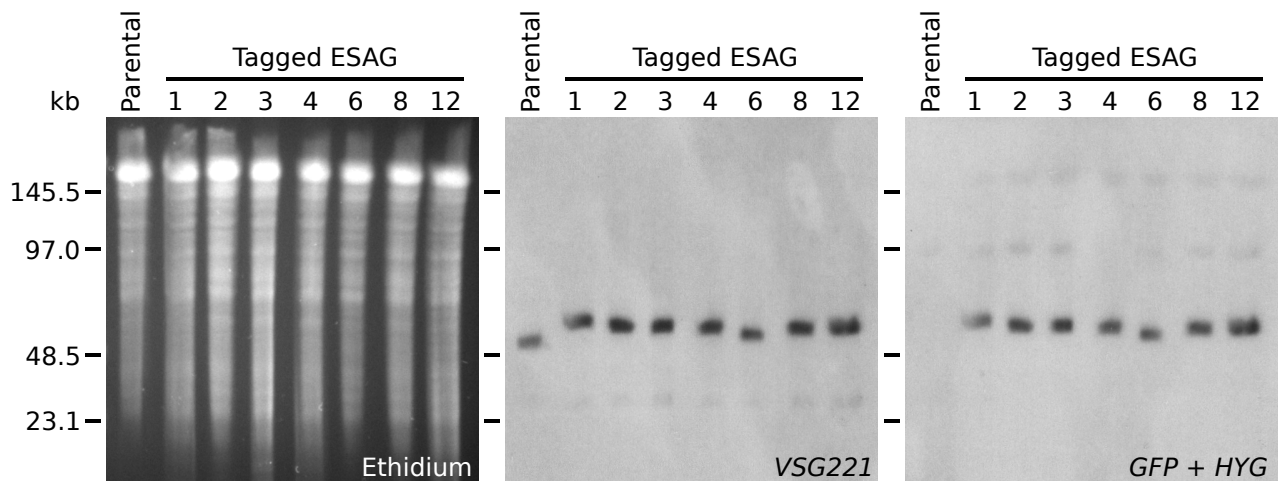

Supplement: Supplemental Data [file supp_M114.047647_mcp.M114.047647-6.pdf]

# Supplemental Figure 6

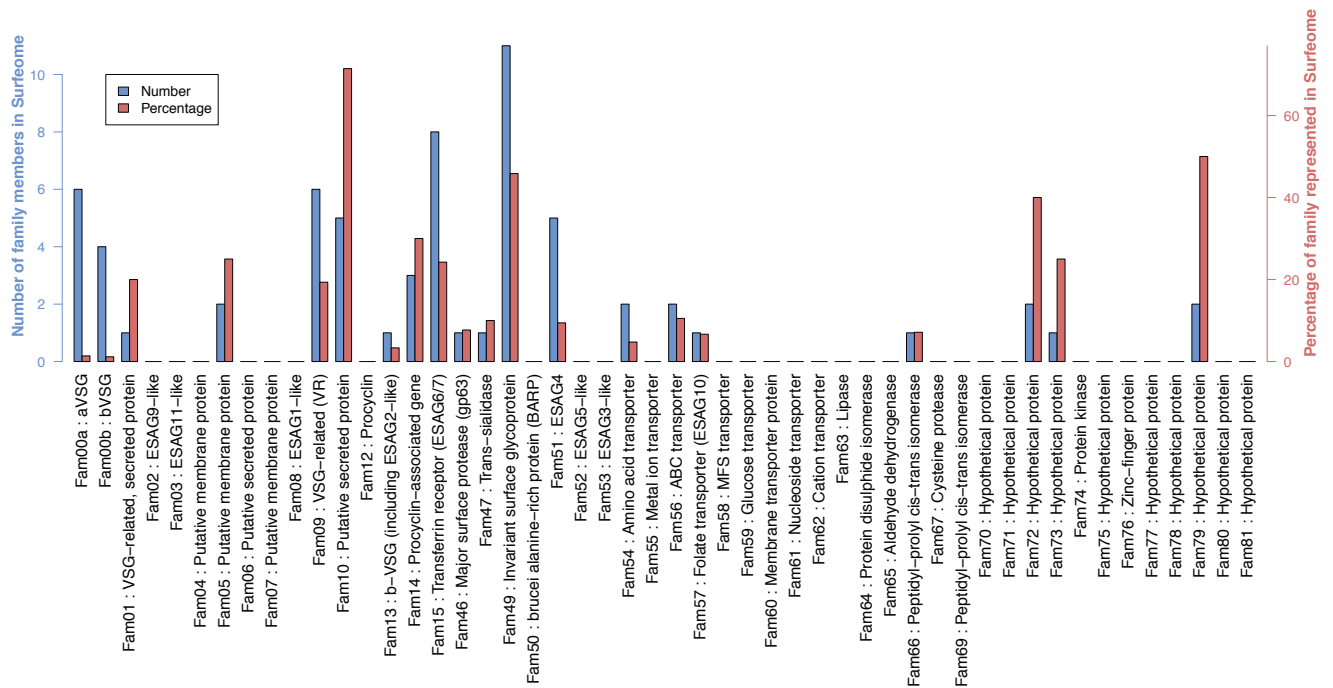

Supplement: Supplemental Data [file supp_M114.047647_mcp.M114.047647-7.pdf]
